# Supplementary material for: The tectonic complex regulates membrane protein composition in the photoreceptor cilium
Source: Nat Commun. 2023 Sep 13;14:5671. doi: 10.1038/s41467-023-41450-z (PMC10500017; doi:10.1038/s41467-023-41450-z)
Supplement: Supplementary file 1 — Supplementary information [file 41467_2023_41450_MOESM1_ESM.pdf]

The Tectonic Complex Regulates Membrane Protein Composition in the Photoreceptor Cilium

Hanh M. Truong[1], Kevin O. Cruz-Colón[2], Jorge Y. Martínez-Márquez[3], Jason R. Willer[3], Amanda M. Travis[3], Sondip K. Biswas[4], Woo-Kuen Lo[4], Hanno J. Bolz[5,6], and Jillian N. Pearring[3,7,*]

[1] Cellular and Molecular Biology Program, University of Michigan, Ann Arbor, MI, USA

[2] Neuroscience Graduate Program, University of Michigan, Ann Arbor, MI, USA

[3] Department of Ophthalmology and Visual Science, University of Michigan, Ann Arbor, MI, USA

[4] Department of Neurobiology, Morehouse School of Medicine, Atlanta, GA, USA

[5] Senckenberg Centre for Human Genetics, Frankfurt am Main, Germany

[6] Institute of Human Genetics, University Hospital of Cologne, Cologne, Germany

[7] Department of Cell and Developmental Biology, University of Michigan, Ann Arbor, MI, USA

[*]Corresponding author: pearring@umich.edu

This PDF includes Supplemental Figures 1-7 and Supplemental Table 1-2.

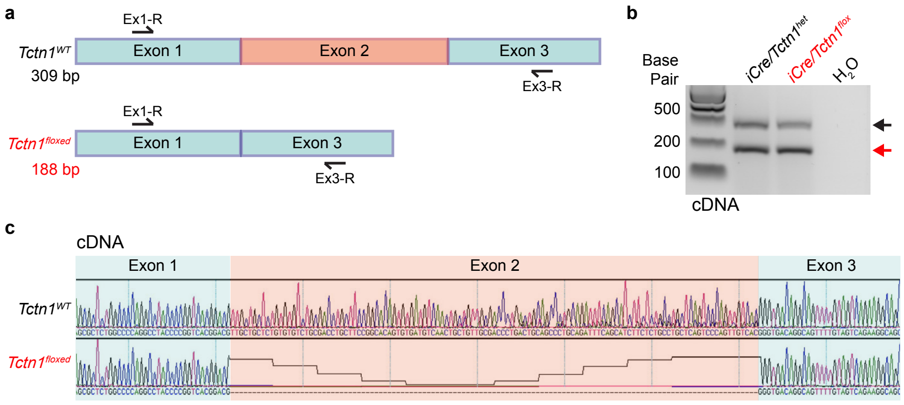

Supplemental Figure 1: Verification of *Tctn1* exon2 excision with addition of cre using Sanger Sequencing.

Sanger sequencing results confirm that *Tctn1* exon2 is excised from cDNA isolated from *iCre/Tctn1*^{het} and *iCre/Tctn1*^{flox} 3 month retinas. (**a**) Diagram showing Tctn1 exon 1-3 with primers used for amplifying the floxed region from cDNA. (**b**) Agarose gel showing bands produced by PCRs from cDNA isolated from *iCre/Tctn1*^{het} and *iCre/Tctn1*^{flox} retinas. Excision of exon 2 only occurs in rod photoreceptors, so 2 bands are produced by the PCR reaction: *Tctn1*^{WT} (black arrow, 309 bp) and *Tctn1*^{Floxed} (red arrow,188 bp). (**c**) Sanger sequencing results show that the *Tctn1*^{floxed} allele has successful excision of exon 2.

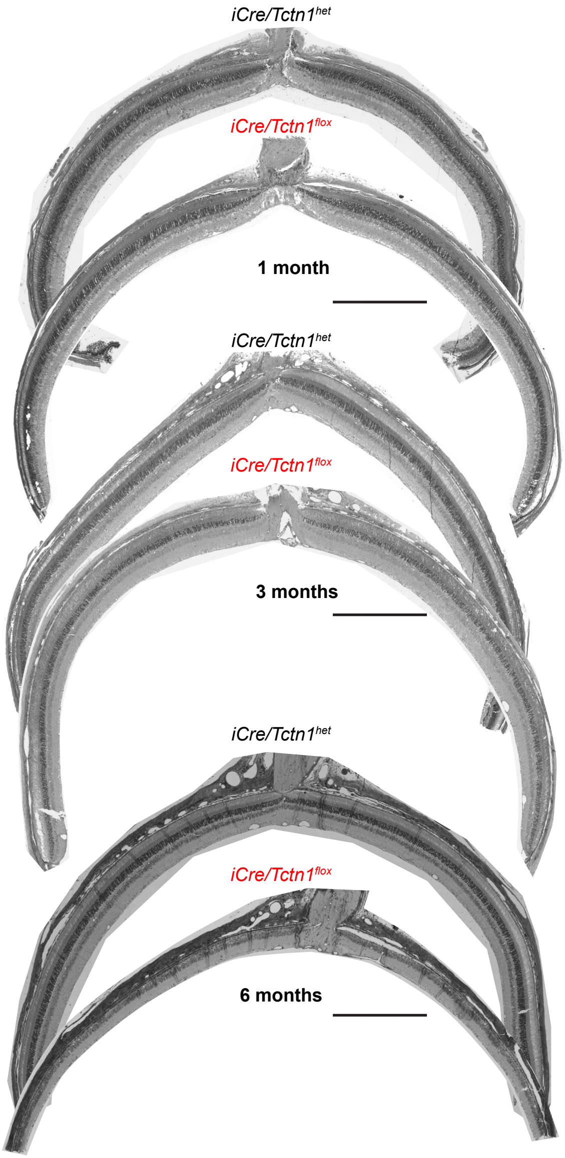

Supplemental Figure 2: Rod photoreceptors of *iCre/Tctn1*^{flox} mice undergo progressive degeneration.

Representative tile scanned light microscope images of entire retinal plastic sections cut through the optic nerve of *iCre/Tctn1*^{het} and *iCre/Tctn1*^{flox} mice aged to 1, 3, and 6 month time points. Scale bars, 500 µm

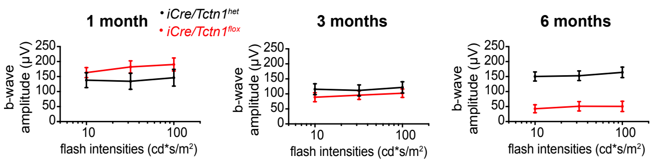

Supplemental Figure 3: Light-adapted cone responses of *iCre/Tctn1^{flox}* mice are affected once rod photoreceptor death has occurred.

Averaged light-adapted b-wave amplitudes from *iCre/Tctn1^{het}* and *iCre/Tctn1^{flox}* mice taken at 1, 3, and 6 month time points were plotted. Error bars indicate S.D.

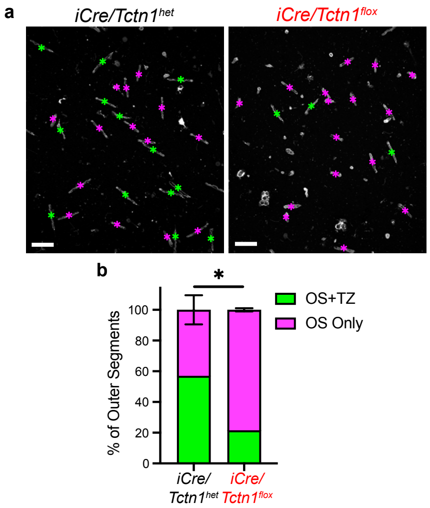

Supplemental Figure 4: Isolated outer segments from *iCre/Tctn1^{het}* and *iCre/Tctn1^{flox}* retinas.

(**a**) Representative images of outer segments isolated from *iCre/Tctn1^{het}* and *iCre/Tctn1^{flox}* mouse retinas stained with WGA (grey). Green asterisk indicates intact outer segments, magenta asterisk indicates broken outer segments. Scale bars, 20 µm (**b**) Bar graph showing the percent of intact outer segment (OS+TZ) and broken outer segments (OS only). n=3 biological replicates for each genotype. Error bars indicate S.D. There is a significant decrease in intact outer segments in the *iCre/Tctn1^{flox}* preparations. Statistical significance was determined with an unpaired two-tailed t test. *, p=0.0219. Source data are provided as a Source Data file.

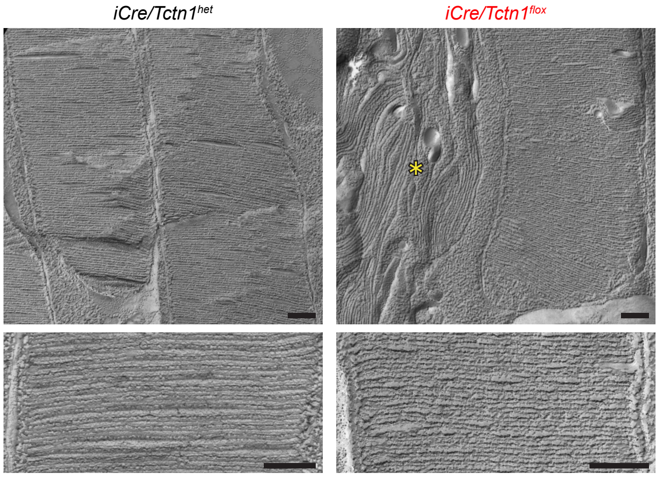

Supplemental Figure 5: Disc structure is normal in *iCre/Tctn1^{flox}* outer segments.

Freeze-fracture electron microscopy images highlighting the disc spacing and structure of *iCre/Tctn1^{het}* and *iCre/Tctn1^{flox}* outer segments at 3 months. Low magnification at the top; high magnification at the bottom. Yellow asterisk indicates disorganized outer segment found in apoptotic rod photoreceptors in *iCre/Tctn1^{flox}* retinas. Scale bars, 200 nm.

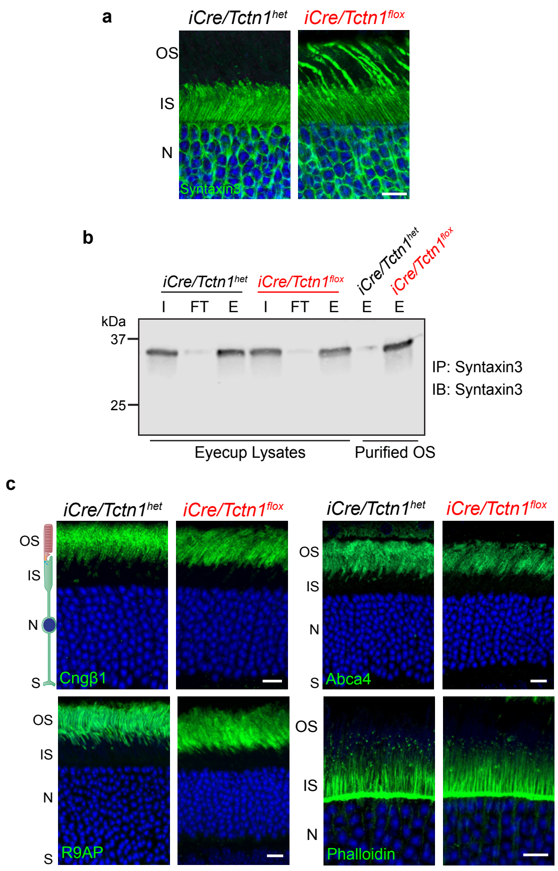

Supplemental Figure 6: Analysis of *iCre/Tctn1^{flox}* retina phenotypes at 1 and 3 months.

(**a**) Representative immunofluorescence images of *iCre/Tctn1^{het}* and *iCre/Tctn1^{flox}* retinal cross sections at 1 month stained with respective inner segment proteins: syntaxin3 (green). Nuclei are counterstained with DAPI (blue). Scale bars, 10 μm. (**b**) Representative Western blot showing endogenous syntaxin 3 immunoprecipitation from *iCre/Tctn1^{het}* and *iCre/Tctn1^{flox}* eyecup lysates and purified outer segment protein lysates. Abbreviations: input (I), flow-through (FT), eluate (E). (**c**) Representative immunofluorescence images of 3 month *iCre/Tctn1^{het}* and *iCre/Tctn1^{flox}* retinal cross sections stained with respective outer segment proteins: Cngβ1, R9AP, Abca4, and actin organizing center, phalloidin (green). Nuclei are counterstained with DAPI (blue). Scale bars, 10 μm.

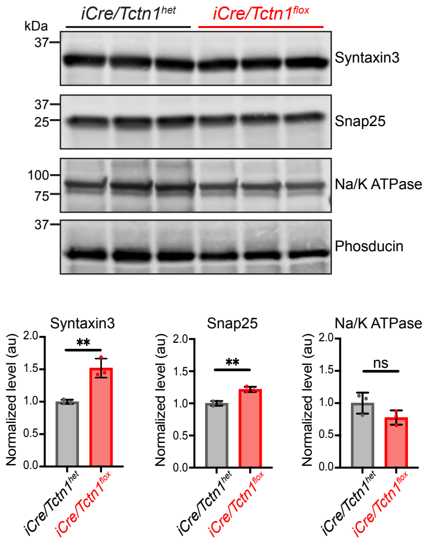

Supplemental Figure 7: Inner segment protein levels in 3 month *iCre/Tctn1^flox^* eyecup lysates. Representative Western blot showing endogenous syntaxin 3, Snap25 and Na/K ATPase levels in *iCre/Tctn1*^{het} and *iCre/Tctn1*^{flox} eyecup lysates at 3 months. Levels of each protein were normalized to phosducin, a soluble protein that is expressed in mouse photoreceptors, to account for ongoing photoreceptors cell loss occurring in *iCre/Tctn1*^{flox} at this age. Bar graphs below show the normalized levels for each protein for n=3 biological replicates. Error bars indicate S.D. Statistical significance was determined with an unpaired two-tailed t test. Syntaxin3 **, p=0.0039; Snap25 **, p=0.0021; Na/K ATPase ns, p=0.1192. Source data are provided as a Source Data file.

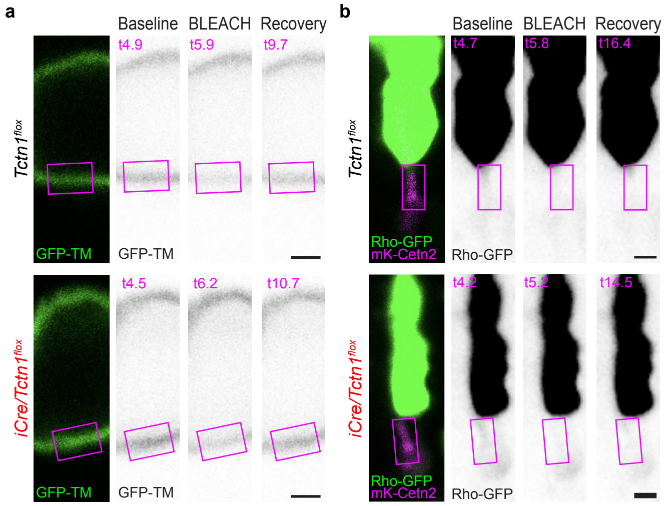

Supplemental Figure 8: Images of GFP-TM and Rho-GFP FRAP.

(**a**) Representative FRAP images from GFP-TM expression at the plasma membrane surrounding the cell body of *Tctn1*^{flox} and *iCre/Tctn1*^{flox} mouse rods. (**b**) Representative FRAP images from Rho-GFP expression at the transition zone of *Tctn1*^{flox} and *iCre/Tctn1*^{flox} mouse rods. For both constructs, high contrast GFP images from the timelapse are shown for baseline, bleach, and recovery with specific digital time (t, seconds) shown at top left**.** Magenta box show ROI used for photobleaching the plasma membrane of transfected rods. Scale bars, 1 μm.

Supplemental Figure 6

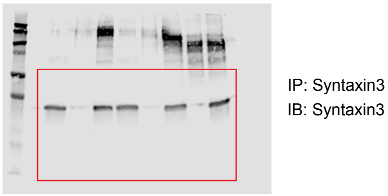

Supplemental Figure 7

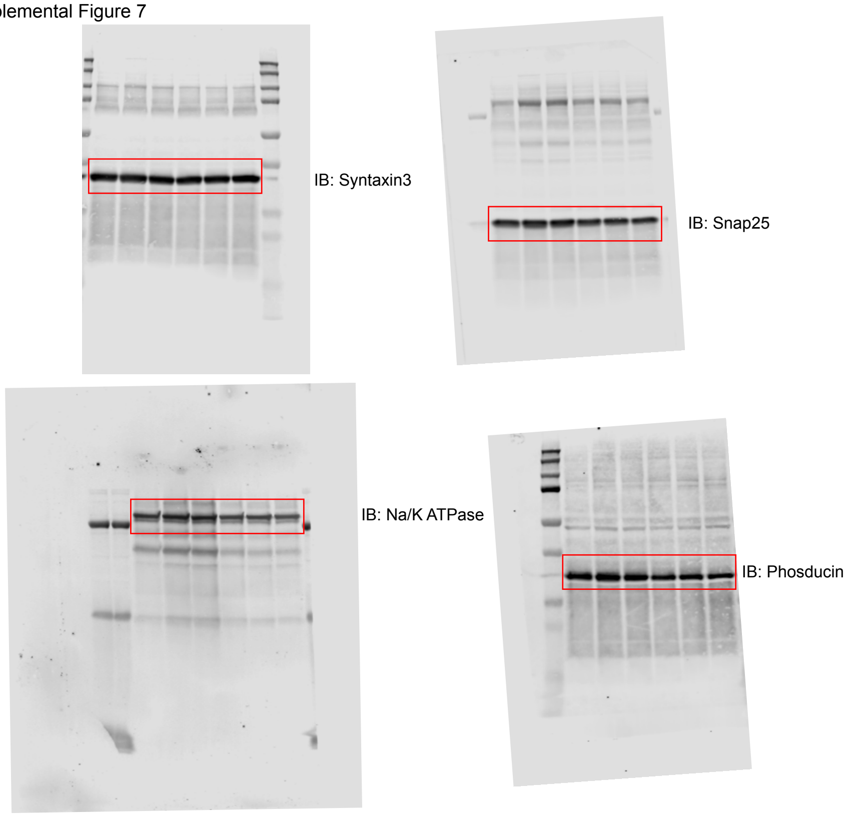

Supplemental Figure 9: **Raw Western blot images**

Supplementary Table 1: Primer sequences and their uses.

Primer Name	Sequence from 5'-3'	Use
mTctn1-Exon2 F	CTGCCAACAGTCCTTCCTTG	For genotyping Tctn1Flox
mTctn1-Exon2 R	CAGGTCGCAGACACACAGAG	For genotyping Tctn1Flox
iCre75 F	TCAGTGCCTGGAGTTGCGCTGTGG	For genotyping rod-specific cre (iCre75)
iCre75 R	CTTAAAGGCCAGGGCCTGCTTGGC	For genotyping rod-specific cre (iCre75)
mTctn1-qPCR-Ex2-F	CTGCTCAGTCCCAGTTGTCA	Used to verify excision of Exon2 of *Tctn1* in mouse cDNA
mTctn1-qPCR-Ex2-R	ATGGATGCAGAAGACGGAAG	Used to verify excision of Exon2 of *Tctn1* in mouse cDNA
mTCTN1 seqF1	ACGTTGCTGCTCTGTGTGTC	Sequencing primers for mouse *Tctn1* cDNA
mTCTN1 seqF2	TTTATTCAGCATGGCACCAA	Sequencing primers for mouse *Tctn1* cDNA
mTCTN1 seqR1	ATGGATGCAGAAGACGGAAG	Sequencing primers for mouse *Tctn1* cDNA
mTCTN1 seqR2	CTGACAGCCAGACTGCACAT	Sequencing primers for mouse *Tctn1* cDNA
Age-mTCTN1 F	GGATCCACCGGTATGGGGTCGCGGGGTCTCCCG	Clone mouse *Tctn1* into pRho plasmid
mTCTN1-myc-NotI R	CTCTTCTGAGATAAGCTTTTGTTCAGCCACAAACGGGAAGAAGAAG	Add C-terminal myc tag to mouse *Tctn1* for pRho plasmid
Age-mKate2-F	ggatccaccggtGCCGCCACCatggtgagcgagctgattaag	Add N-terminal mKate tag to mouse *Cetn2* for pRho plasmid
mKate-Centrin2-R	CCATAGATCTGAGTCCGGAtctgtgccccagtttgctagg	Add N-terminal mKate tag to mouse *Cetn2*
mKate-Centrin2-F	agcaaactggggcacagaTCCGGACTCAGATCTATGGC	Add N-terminal mKate tag to mouse *Cetn2*
NotI-Centrin-R	ggagtgcggccgcTTAATAGAGGCTGGTCTTTTTCATGATGCG	Add N-terminal mKate tag to mouse *Cetn2* for pRho plasmid
SalI-hRK-F	GTCGACGGGCCCCAGAAGCCTGGTG	Replacing bovine rhodopin promoter with human rhodopsin kinase promoter + SV40 SD/SA
hRK-Kpn1-R	GCCCGCGGTACCCGGCGGGTACAATTCCGCAGC	Replacing bovine rhodopin promoter with human rhodopsin kinase promoter + SV40 SD/SA

Supplementary Table 2: Antibodies used, their sources, and recommended dilutions.

Name	Source	Catalog #	Dilution for Hard-fixed Eyecup IF	Dilution for Isolated Outer Segment TZ IF	Dilution for Fresh Retina TZ IF	Dilution for WB
Abca4 (Gt)	Everest	EB08615	1:1000	NA	NA	NA
Cngβ1 (Rb)	Steven Pittler Lab	NA	1:1000	NA	NA	NA
Multi Ubiquitin (M)	MBL International	D058-3	1:1000	NA	NA	NA
Myc (Rb)	Cell Signaling	2278S	1:1000	NA	1:100	NA
Na/K ATPase (M)	Santa Cruz	SC58628	1:1000	NA	NA	1:5000
Peripherin (Sh)	Gabriel Travis Lab	NA	1:1000	NA	NA	NA
Phalloidin-iFluor 647	Abcam	ab176759	1:1000	NA	NA	NA
R9AP (Rb)	Vadim Arshavsky Lab	NA	1:1000	NA	NA	NA
Rhodopsin-1D4 (M)	Abcam	ab5417	1:1000	NA	NA	NA
Snap25 [SMI81] (M)	BioLegend	836304	1:1000	NA	NA	1:2000
Syntaxin3 (Rb)	Proteintech	15556-1-AP	1:1000	NA	NA	1:2000
Flag M2 (M)	Sigma-Aldrich	F1804-5MG	1:2000	NA	NA	NA
WGA-Alexa 594	Thermo Fisher	W11262	1:2000	NA	NA	NA
B9d1 (Rb)	Jeremy Reiter Lab	NA	NA	1:100	NA	NA
Spata7 (Rb)	Rui Chen Lab	NA	NA	1:100	NA	NA
Alpha Tubulin (M)	Sigma-Aldrich	T9026	NA	1:1000	NA	NA
Centrin1 (M)	EMD Millipore	04-1624	NA	1:1000	1:100	NA
Cep290 (Rb)	Bethyl laboratories	A301-659A	NA	1:1000	1:50	NA
Bbs5 (M)	Clay Smith Lab	NA	NA	1:200	NA	NA
ΔC2Tubulin (Rb)	Ryoma Ohi Lab	NA	NA	1:2000	NA	NA
Ahi (Rb)	Joseph Gleeson Lab	NA	NA	1:250	NA	NA
Rpgr (Rb)	Proteintech	16891-1-AP	NA	1:500	NA	NA
Phosducin (Sh)	Vadim Arshavsky Lab	NA	NA	NA	NA	1:5000
Rom1 (Sh)	Vadim Arshavsky Lab	NA	NA	NA	1:50	NA
