# Supplementary material for: The tectonic complex regulates membrane protein composition in the photoreceptor cilium
Source: Nat Commun. 2023 Sep 13;14:5671. doi: 10.1038/s41467-023-41450-z (PMC10500017; doi:10.1038/s41467-023-41450-z)
Supplement: Supplementary file 3 — Description of Additional Supplementary files [file 41467_2023_41450_MOESM3_ESM.docx]

**Supplemental Data 1: Tandem-mass tagging mass spectrometry data from *iCre/Tctn1^het^,* *iCre/Tctn1^flox^*, and *C57Bl6J* 3-month purified outer segments.**
